# Antifungal Properties of Bioactive Compounds Isolated from *Fucus vesiculosus* Supercritical Carbon Dioxide Extract

**DOI:** 10.3390/molecules29245957

**Published:** 2024-12-17

**Authors:** Katarzyna Tyśkiewicz, Felix Rüttler, Renata Tyśkiewicz, Artur Nowak, Marcin Gruba, Anita Wziątek, Agnieszka Dębczak, Michał Sandomierski, Walter Vetter

**Affiliations:** 1 Department of Food Chemistry (170B), Institute of Food Chemistry, University of Hohenheim, Garbenstraβe 28, D-70599 Stuttgart, Germany; kasiawest@yahoo.com (K.T.); felix.ruettler@uni-hohenheim.de (F.R.); 2 Analytical Laboratory, Łukasiewicz Research Network—New Chemical Syntheses Institute, Al. Tysiąclecia Państwa Polskiego 13A, 24-110 Puławy, Poland; renata.tyskiewicz@ins.lukasiewicz.gov.pl; 3 Department of Industrial and Environmental Microbiology, Institute of Biological Sciences, Faculty of Biology and Biotechnology, Maria Curie–Skłodowska University, Akademicka 19, 20-033 Lublin, Poland; artur.nowak@mail.umcs.pl; 4 Supercritical Extraction Research Group, Łukasiewicz Research Network—New Chemical Syntheses Institute, Al. Tysiąclecia Państwa Polskiego 13A, 24-110 Puławy, Poland; marcin.gruba@ins.lukasiewicz.gov.pl (M.G.); anita.wziatek@ins.lukasiewicz.gov.pl (A.W.); agnieszka.debczak@ins.lukasiewicz.gov.pl (A.D.); michal.sandomierski@ins.lukasiewicz.gov.pl (M.S.)

**Keywords:** separation, carbon dioxide, enzyme, biostimulant, fatty acid, fucosterol

## Abstract

The exploration of natural antifungal substances from algal origins is significant due to the increasing resistance of pathogens to conventional antifungal agents and the growing consumer demand for natural products. This manuscript represents the inaugural investigation into the antifungal attributes of bioactive compounds extracted from *Fucus vesiculosus* via supercritical carbon dioxide (scCO_2_) extraction utilizing contemporary countercurrent chromatography (CCC). In aligning with the prospective utilization of this extract within the agricultural sector, this study also serves as the preliminary report demonstrating the capability of *Fucus vesiculosus* scCO_2_ extract to enhance the activity of plant resistance enzymes. The fractions obtained through CCC were subjected to evaluation for their efficacy in inhibiting the macrospores of *Fusarium culmorum*. The CCC methodology facilitated the successful separation of fatty acids (reaching up to 82.0 wt.% in a given fraction) and fucosterol (attaining up to 79.4 wt.% in another fraction). All CCC fractions at the concentration of 1.0% were found to inhibit 100% of *Fusarium culmorum* growth. Moreover, *Fucus vesiculosus* scCO_2_ extract was able to activate plant resistance enzymes (Catalase, Ascorbic Peroxidase, Guaiacol Peroxidase, Phenylalanine Ammonia-Lyase, and Phenylalanine Ammonia-Lyase Activity).

## 1. Introduction

Due to the escalating resistance to pharmacological agents, such as antibiotics, there is a growing inquiry for novel natural resources exhibiting antimicrobial, or in this case, antifungal, properties. Furthermore, such resources typically possess significantly diminished toxicity in comparison to antibiotics and conventional herbicides. The global algae valorization was estimated to reach EUR 11.8 billion in 2030 [1]. The European Institute of Innovation and Technology (EIT) qualified algae biomass as a novel food to incorporate into dietary supplements [2]. One of the potentially interesting organisms in this context is the brown algae *Fucus vesiculosus* (which belongs to *Fucaceae*, and is common in the Irminger Sea, Norwegian Sea, Barents Sea, and White Sea zones), which was found to have several medical benefits, including antioxidant, anti-obesity, anti-inflammatory, antimicrobial (antiviral), anticancer, anti-aging [3,4,5,6,7], and antitumor properties [8,9,10], as well as antihyperglycemic and anti-hypercholesterolemic properties of alginic acid [11,12,13,14,15].

Most investigations focused on water extracts of *Fucus vesiculosus* as fractions of certain compounds (phenolic compounds, fucoidans, phlorotannins, and water-soluble vitamins [16,17] with the potential to be used as a biostimulant agent [18,19,20,21] and an antioxidant component of skin care emulsions [22]. However, also the pattern of nonpolar compounds is promising as the seaweed was found to be rich in sterols (10.6 wt.%), chlorophylls and xanthophylls (15.1 wt.%), glycolipids (29.9 wt.%), phospholipids (6.5 wt.%), free fatty acids (4.2 wt.%), triacylglycerols (11.8 wt.%) [23,24], and fat-soluble vitamins [16,17,25,26]. For instance, Lorenzo et al. identified 24 fatty acids in the extract obtained from the mixture of CHCl_3_/CH_3_OH/H_2_O (1:2:0.8, *v*/*v*/*v*) [27]. Previous studies with polar solvents (alcoholic extracts) indicated the antifungal properties of *Fucus vesiculosus* [28]. However, the mechanism of bioactive compounds’ effect on phytopathogenic fungi has not been discovered yet. A positive role of sterols has been suggested by Newman et al. [29], but this was not confirmed in another study [30].

Interestingly, the enrichment of non-polar valuable compounds from *Fucus vesiculosus* can be carried out using carbon dioxide in a supercritical state (scCO_2_) [31,32,33], which also enabled to quantify fucosterol at the level of 8.1 wt.% [31]. In addition, Tyśkiewicz et al. [31] observed that the scCO_2_ extract of *Fucus vesiculosus* inhibited 100% of the growth of *Fusarium culmorum* and *Fusarium oxysporum* macroconidia within 144 h. However, the origins could not be determined on a molecular level.

The goal of the present investigation was to fractionate a scCO_2_ extract of *Fucus vesiculosus* to get more insights into the bioactive compounds responsible for the previously reported effect. For this purpose, countercurrent chromatography (CCC) was employed for the separation of bioactive compounds [34,35,36,37]. CCC is an instrumental method that utilizes a biphasic solvent system and separates compounds according to their partition coefficients in the two phases [38,39]. CCC allows us to inject and separate ~1 g samples, while the omission of solid support eliminates sample loss through irreversible adsorption and the degradation of sensitive analytes.

Therefore, CCC is well-suited for the fractionation of sample extracts [35,40] which then can be analyzed using analytical methods (here: GC/FID (gas chromatography equipped with flame ionization detection), and UPC^2^ (ultra-performance convergence chromatography)), and also applied to determine antifungal properties against *Fusarium culmorum*. CCC has been previously used to fractionate bioactive compounds from scCO_2_ extracts [41,42,43]. Additionally, *Fucus vesiculosus* scCO_2_ extract was tested to activate plant resistance enzymes.

## 2. Results and Discussion

### 2.1. Fatty Acids and Fucosterol Content in Fucus vesiculosus Biomass and scCO_2_ Extract

*Fucus vesiculosus* is distinguished by a significant content of unsaturated fatty acids, which constitute approximately 72 wt.% of its overall fatty acid profile (Table 1). The biomass of this macroalga encompasses essential fatty acids such as linoleic, γ-linolenic, arachidonic, and eicosapentaenoic acid. Predominant fatty acids that are recognized include palmitic, oleic, and linoleic acid, with the overall lipid content accounting for 1.8 wt.% of the dry weight. Comparable proportions of unsaturated fatty acids (approximately 75 wt.%) were reported in *Fucus vesiculosus* by Herbreteau et al. [44], suggesting that the presence of these fatty acids enhances the species’ potential for commercial applications and its biochemical functions in the regulation of cellular membranes. Nevertheless, the amount of fatty acids present in *Fucus vesiculosus*, as investigated by Herbreteau et al. [44], was found to be two times lower (1.36 wt.%) in comparison to the findings of our study. Different extraction methods yield varying contents of fatty acids, influenced by the solvent systems and techniques employed. This variability is due to the differential solubility levels of lipid components and the efficiency of the extraction process in isolating specific lipid classes [45]. In this study, the SFE (scCO_2_) at 50 °C and 500 bar resulted in an extraction yield of 4.6 wt.%. The high pressure was selected since previous studies with related samples (*Fucus evanescens*, *Saccharina japonica,* and *Saccharina oligocystum*) indicated a high extraction yield at 60 °C and 550 bar [46]. Likewise, the extraction yield was higher than in our previous study [31], which provided ~1/3rd less extract (~2.8 wt.%) at 50 °C and 300 bar.

**Figure 1:**
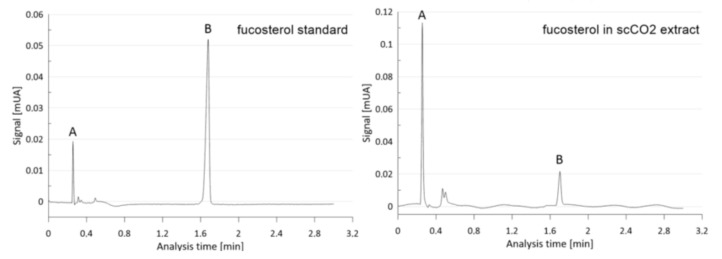
SFC chromatogram (UPC^2^ system) of the *Fucus vesiculosus* scCO_2_ extract (fucosterol standard on the **left**, extract on the **right**) with A–solvent; B–fucosterol.

Recently, Tyśkiewicz et al. [31] reported the presence of fucosterol in *Fucus vesiculosus*. The present sample contained fucosterol at 0.13 wt.% of dry weight (DW) in the biomass and 2.8 wt.% in supercritical carbon dioxide extract of *Fucus vesiculosus* (Figure 1).

**Figure 2:**
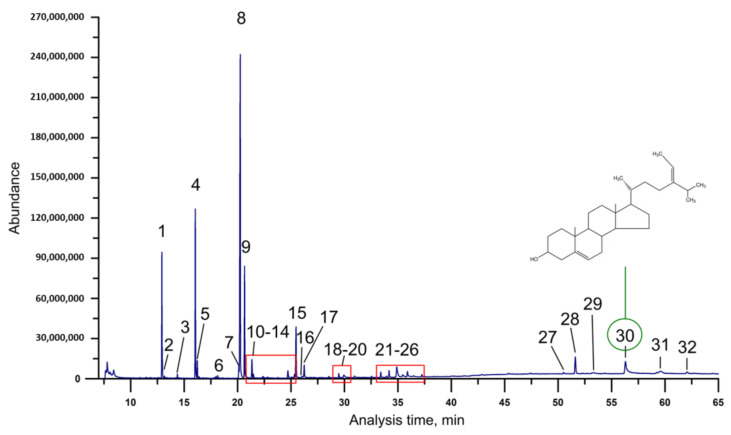
GC/MS-chromatogram (full scan mode) of *Fucus vesiculosus* scCO_2_ extract (numbers according to Table 2).

The assessment of retention indices (RI) was conducted through the introduction of *Fucus vesiculosus* scCO_2_ extract into the gas chromatography–mass spectrometry (GC/MS) apparatus, utilizing predetermined parameters, subsequently followed by the injection of alkanes ranging from C_8_ to C_40_ into the GC/MS system under identical conditions as those employed for the methyl esters (Section 3.3.4). The retention indices corresponding to each methyl ester were calculated utilizing the formula described by Rostad et al. [32]. The result of GC/MS analysis is presented in Table 2 and Figure 2.

The GC/MS analysis revealed the presence of fatty acids and fucosterol in *Fucus vesiculosus* scCO_2_ extract. The obtained mass spectra for each compound was compared with the NIST library. Based on GC/MS analysis, saturated fatty acids were distinguished from unsaturated ones by the absence of the fragment ions of 74, 87, and 143, in mass spectrum in the case of the latter. Fatty acids 14:0 (**1**), 16:0 (**4**), 18:0 (**7**) and 20:0 (**13**) were characterized by the molecular ion *m*/*z* 242, *m*/*z* 270, *m*/*z* 298 and *m*/*z* 326, respectively, with the characteristic fragment ion of *m*/*z* 211, *m*/*z* 239, *m*/*z* 267, and *m*/*z* 295, respectively, as the effect of methoxy group loss [M-31]^+^, confirming that the form of fatty acids was that of methyl esters. In turn, fatty acids 18:1*n-9* (**8**), 18:2*n-6* (**9**), and 18:3*n-6* (**11**) exhibit an analogous pattern with the fragment ion *m*/*z* 55. Nonetheless, the fragment ions that facilitate the comparative analysis of these three compounds were as follows: *m*/*z* 265 for oleic acid methyl ester, *m*/*z* 263 for linoleic acid methyl ester, and *m*/*z* 261 for γ-linolenic acid methyl ester [52]. The identification of fucosterol (**30**) was based on the molecular mass of *m*/*z* 412 with the characteristic fragment ion *m*/*z* 314.

### 2.2. Fractionation of the scCO_2_ Extract with Countercurrent Chromatography (CCC)

Since the main interest was the verification of the bioactivity of fucosterol which was previously identified in an scCO_2_ extract of *Fucus vesiculosus* [31], two subsequent CCC methods were applied. In the first step, CCC was operated in co-current mode (ccCCC) using *n*-hexane/ethanol/methanol/water (34:11:12:2, *v*/*v*/*v*/*v*) as the solvent system (Section 3.3.2). In co-current CCC, the elution of analytes is accelerated, resulting in a fast elution of all compounds at the dispense of the resolution [53,54,55]. Therefore, this “pre-run” was mainly used to separate undesired, more polar compounds. Under stable conditions (no loss of stationary phase during the run), the amount of the mobile phase corresponds with the volume displaced during equilibration. This was 24 mL in the present case (S_f_ 90%), so there was ~200 mL of stationary phase in the coils. Once this volume of stationary phase is eluted, no more sample can be in the coil. At a flow rate of 4 mL/min of the stationary phase, this point was reached after a run time of 50 min. Two ccCCC runs were subsequently injected with ~0.8 g scCO_2_ extract (total amount used: 1.6 g) each. Fraction 1 (ccCCC fraction 1) was gained from 0–37.5 min and Fraction 2 (ccCCC fraction 2) from 37.5–50 min. Due to the high contribution of the nonpolar esterified fatty acids (triacylglycerols), the resulting weight of the nonpolar ccCCC Fraction 1 containing the quantitative amount of fucosterol was >90% of the sample. However, this approach simplified the subsequent conventional CCC in good time without the necessity to empty the coil system (by blowing out or elution/extrusion). This approach enabled us to perform repeated CCC runs with the same system.

In this project, ~500 mg of ccCCC Fraction 1 was used for the conventional CCC (Section 3.3.2). The separation was performed with the BTF system due to previous good experience in the field of sterols [40,56]. To separate the bulk of the triacylglycerols, a long pre-run (Fraction CCC pre-run) was collected and the fractionation into 15 CCC fractions (CCC Fraction 1–15) was started after 102 min. In agreement with expectations, the bulk of the sample was detected in the pre-run (347 mg, which corresponded with >50% of the injected sample weight. Due to the pre-separation of the bulk of the triacylglycerols in the pre-run, the weight in the collected fractions was comparably low and ranged between 1.5 mg (CCC fraction 14) and 7.7 mg (CCC fraction 6). UPC^2^ analysis of samples enabled the detection of fucosterol in CCC Fraction 7–14 (with the highest abundance in CCC Fraction 9–11; ~70% of the compound; total 6.7 mg; 79.4 wt.% fucosterol in CCC Fraction 11). However, subsequent analysis of fatty acids (after transmethylation of aliquots) indicated that fatty acids were present in all CCC fractions (Table 3). The highest amount of fatty acids was detected in the fraction CCC post-run (82.0 wt.%), but during the fractionation, the contributions decreased. CCC Fraction 11, which was richest in fucosterol (see above), contained 21.8 wt.% fatty acids, and CCC Fraction 10 contained 23.8 wt% fatty acids. Accordingly, the fatty acids were the only or main impurities of fucosterol. Subsequently, all fractions were subjected to microbiological investigations involving the *Fusarium culmorum* 1913 pathogen.

### 2.3. Antifungal Properties of CCC Fractions

**Figure 3:**
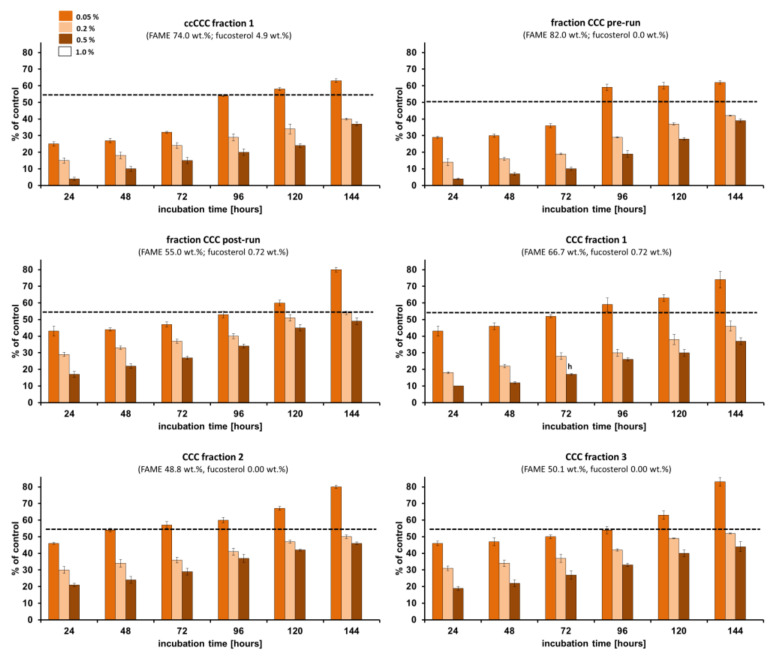
The percentage of germinated macroconidia of *Fusarium culmorum* 1913 treated with *Fucus vesiculosus* scCO_2_ CCC fractions (ccCCC Fraction 1, CCC pre-run, CCC post-run, CCC Fraction 1–6). Bars represent standard deviations (SD).

**Figure 4:**
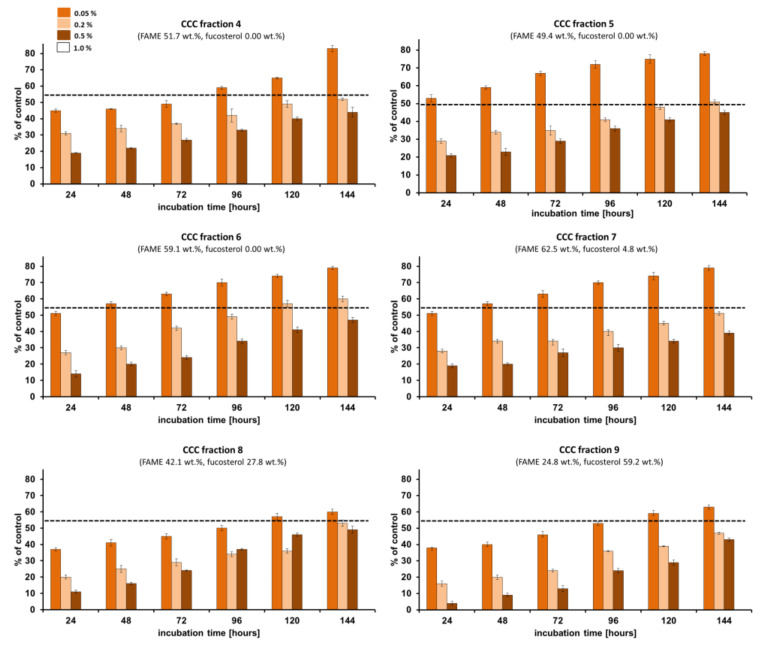
The percentage of germinated macroconidia of *Fusarium culmorum* 1913 treated with *Fucus vesiculosus* scCO_2_ CCC fractions (CCC Fraction 4–9). Bars represent standard deviations (SD).

**Figure 5:**
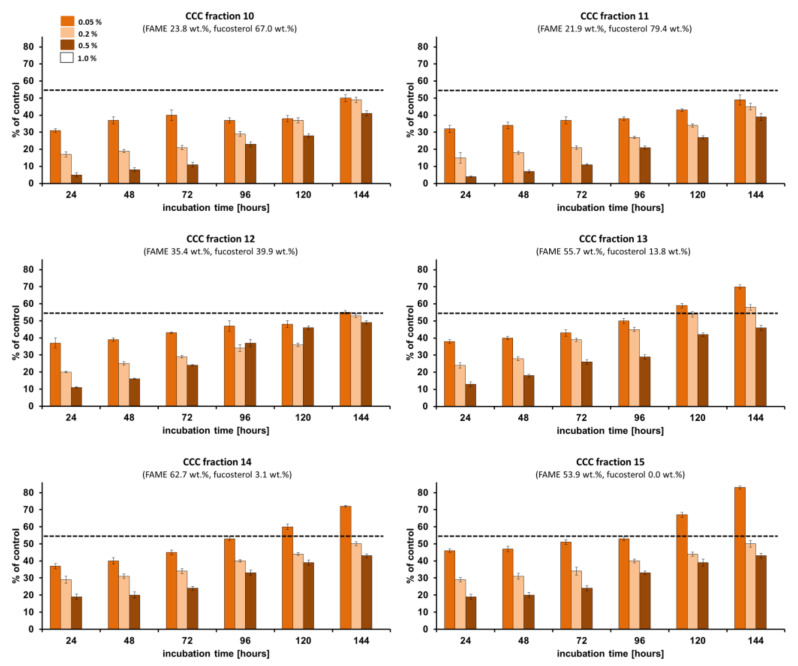
The percentage of germinated macroconidia of *Fusarium culmorum* 1913 treated with *Fucus vesiculosus* scCO_2_ CCC fractions (CCC Fraction 10–15). Bars represent standard deviations (SD).

The zero percentage of fungal macroconidia germination (the lowest percentage has the better inhibition effect) was observed when the phytopathogen (*Fusarium culmorum* 1913) was treated with *Fucus vesiculosus* scCO_2_ CCC fractions at the concentration of 1.0%, and these results will be discussed in the following text. All of the studied CCC fractions at the concentration of 1% were able to inhibit 100% of *Fusarium culmorum* macroconidia growth. The percentage of germinated *Fusarium culmorum* 1913 macroconidia was lower than 50% when treated with all studied CCC fractions at the concentration of 0.5% after 24–144 h of incubation (Figure 3, Figure 4 and Figure 5). Almost all CCC fractions at the concentration of 0.2% studied against *Fusarium culmorum* resulted in the percentage of germinated *Fusarium culmorum* 1913 macroconidia lower than 50% after 120 h of incubation and then slightly increased to 51–60% after the next 24 h of incubation (maximum in CCC Fraction 6). Only after the implementation of CCC Fraction 10 and CCC Fraction 11 at the concentration of 0.05% on *Fusarium culmorum* did the percentage of germinated macroconidia not exceed 50% after 144 h of incubation.

The lowest percentage of germinated *Fusarium culmorum* macroconidia (4–5%) after 24 h of incubation was observed when CCC Fraction 9–11, ccCCC Fraction 1, and the Fraction CCC pre-run were applied at the concentration of 0.5%. The application of those fractions at the concentration of 0.2% on *Fusarium culmorum* resulted in 14–17% of germinated macroconidia and at the concentration of 0.05%, the germination of *Fusarium culmorum* macroconidia was higher (38%, 31%, 32%, 25%, 29%, respectively, for CCC Fraction 9–11, ccCCC Fraction 1, and the fraction CCC pre-run). The lowest germination of *Fusarium culmorum* macroconidia after 144 h of incubation was analyzed for ccCCC Fraction 1 (37%) at the concentration of 0.5%, ccCCC Fraction 1 (40%) at the concentration of 0.2%, and CCC Fraction 11 (49%) at the concentration of 0.05%. The highest germination of *Fusarium culmorum* macroconidia after 144 h of incubation was observed for CCC Fraction 8 and CCC Fraction 12 (49%) at the concentration of 0.5%, CCC Fraction 6 (60%) at the concentration of 0.2% and CCC Fraction 2, CCC Fraction 3, and CCC Fraction 15 (83%) at the concentration of 0.05% (Figure 3, Figure 4 and Figure 5).

CCC Fractions 9–11 of *Fucus vesiculosus,* which were richest in fucosterol (59.2 wt.%, 67.0 wt.% and 79.4 wt.%, respectively), showed similarly high percentages of germinated spores after 24–144 h of incubation. For a better comparison, the result after 24 h was used, which usually displays the strongest effect (at longer incubation times, *Fusarium* is usually defending itself which leads to a higher percentage of geminates spores at longer incubation time). Specifically, this was 4–43% (CCC Fraction 9), 5–41% (CCC Fraction 10), and 4–39% (CCC Fraction 11) at the fraction concentration of 0.5%.

However, the other CCC fractions (CCC Fractions 1–8 and 12–15 at the concentration of 0.5%) of the scCO_2_ extract of *Fucus vesiculosus* were effective against *Fusarium culmorum* 1913. Actually, similar effects as those seen in the “fucosterol” fractions were also obtained with ccCCC Fraction 1 (4–37% of germinated spores) and the Fraction CCC pre-run (4–39% of germinated spores), which were rich in fatty acids (74.0 wt.% and 82.0 wt.%, respectively) (Figure 3, Figure 4 and Figure 5). At a lower content of fatty acids (66.7 wt.%, CCC Fraction 1), the percentage of germinated *Fusarium culmorum* macroconidia was 2.5 times higher (10%) compared to ccCCC Fraction 1 (4%) after 24 h of incubation (CCC fractions at the concentration of 0.5%). In this context, it should be noted that fatty acids were determined after conversion into FAMEs so that the initial binding form was unknown. In tail-to-head mode (upper phase mobile), non-polar compounds are eluted first. Accordingly, the effect was mostly linked to triacylglycerols. Algae fatty acids were shown to display anti-inflammatory, antiproliferative, anticancer, antibacterial, antiviral, antitumor effects [46,57,58,59], and antifungal properties against *Fusarium* species, which had not been reported in the literature.

**Figure 6:**
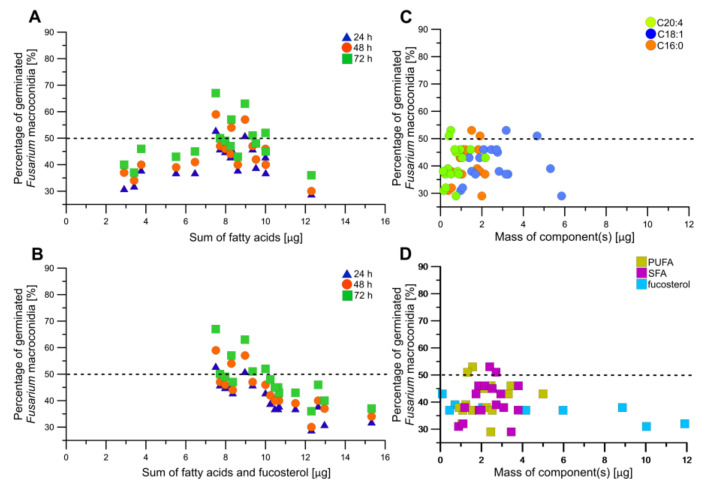
The influence of particular component(s) (**A**,**B**) on *Fusarium culmorum* 1913 germination after 24–144 h of incubation ((**C**,**D**) after 24 h of incubation).

When evaluating the data exclusively concerning only the sum of fatty acids in obtained CCC fractions, it becomes evident that both the minimal and the maximal concentrations of fatty acids had an influence on the reduction of *Fusarium culmorum* macroconidia inhibition (Figure 6A). It may be explained by the synergistic effect of other non-polar compounds potentially present in obtained CCC fractions, such as fat-soluble vitamins [60], carotenoids [61] and chlorophylls [62], which have been found to demonstrate significant antifungal capabilities investigated in various studies, ranging from direct antimicrobial properties to their involvement in disease identification and the mechanisms of plant defense [63]. In turn, an elevated concentration of fatty acids along with fucosterol resulted in a notable reduction in the percentage of *Fusarium culmorum* macroconidia growth, thereby indicating that the suppression of *Fusarium culmorum* growth was greater than in the case of fatty acids alone (Figure 6B). These results confirmed previous treatments of *Fusarium culmorum* Fc37 and *Fusarium culmorum* CBS122 macroconidia with an unfractionated scCO_2_ extract of *Fucus vesiculosus* [31] and indicated that fucosterol may play an important role in this context. Fucosterol was found before to act as an antifungal agent against certain phytopathogens (*Pyricularia oryzae*, *Staphylococcus epidermidis*, *Aspergillus niger*, *Candida albicans*, *Escherichia coli*, *Staphylococcus aureus*, *Pseudomonas aeruginosa*, *Fusarium culmorum*) and yeast species (*Curvularia lunata*, *Stachybotrys atra*, *Microsporum canis*) and the mode of action was supposed to be inhibiting fungal growth by causing structural degradation in fungal cells [24,31,64].

Unsaturated fatty acids, such as those involved in the action of MBX-7591, have shown potential in combating triazole-resistant strains of *Aspergillus fumigatus* by altering membrane saturation and integrity. This highlights their potential in treating resistant filamentous fungi [65]. However, Liu et al. [66] reported saturated fatty acids, specifically 4:0, 6:0, 8:0, 10:0, 12:0, and 16:0 to exhibit more pronounced antifungal properties against *Fusarium oxysporum* compared to the unsaturated fatty acids, such as 18:1*n-9*. The lowest percentage of germinated *Fusarium culmorum* macroconidia (25–31%) after 24 h of incubation was in accordance with the highest content of 18:1*n-9* (ccCCC Fraction 1, 4.8 μg in 0.5% fraction; Fraction CCC pre-run, 5.8 μg in 0.5% fraction, Figure 6C) as well as the highest content of fucosterol (CCC Fraction 10, 10.1 μg in 0.5% fraction; CCC Fraction 11, 11.9 μg in 0.5% fraction) (Figure 6C,D). A similar effect was reported by Yang et al. [67] for oleic acid against *Candida albicans*. In some cases, higher contents of bioactive compounds may have adverse effects on pathogen growth inhibition like in the case of 16:0, 20:4*n-6*, SFA, and PUFA (Figure 6C,D). For instance, Avalos and Carmen Limón [68] and Naz et al. [69] suggested a lower inhibition percentage of pathogen growth with the increasing content of carotenoids. Bioactive compounds having antioxidant properties may enhance the survival of phytopathogens in the natural environment and protect them against severe conditions (influence of stress and light) [68,69].

On the basis of the obtained results, it may be concluded that a better effect in limiting the growth of fungus was observed when the CCC fraction was characterized by the high content of fatty acids (over 70 wt.%) or when the CCC fraction contained the mixture of fatty acids and fucosterol.

**Figure 7:**
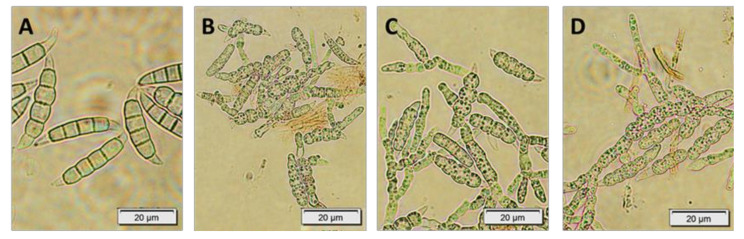
*Fusarium culmorum* 1913 treated with *Fucus vesiculosus* CCC Fraction 11 after 120 h of incubation; fraction concentration of 1.0% (**A**), 0.5% (**B**), 0.2% (**C**) and 0.05% (**D**).

Overall, sterols, which are integral components of plant cell membranes, could disrupt fungal growth and viability by interfering with their sterol biosynthesis and membrane integrity [29]. A microscopic view of *Fusarium culmorum* 1913 macroconidia treated with the *Fucus vesiculosus* scCO_2_ CCC Fraction 11 at the concentration of 0.05–1.0% after 120 h of incubation is presented in Figure 6A–D. *Fusarium* macroconidia growth is primarily facilitated through sporulation or conidiation, which generates large quantities of conidia [70]. In this manner, Figure 7A presents total inhibition of *Fusarium culmorum* macroconidia growth (conidia without hyphae) when treated with 1% of CCC Fraction 11. The more conidia with hyphae, the lower *Fusarium culmorum* growth inhibition, as can be seen in Figure 7B–D.

**Figure 8:**
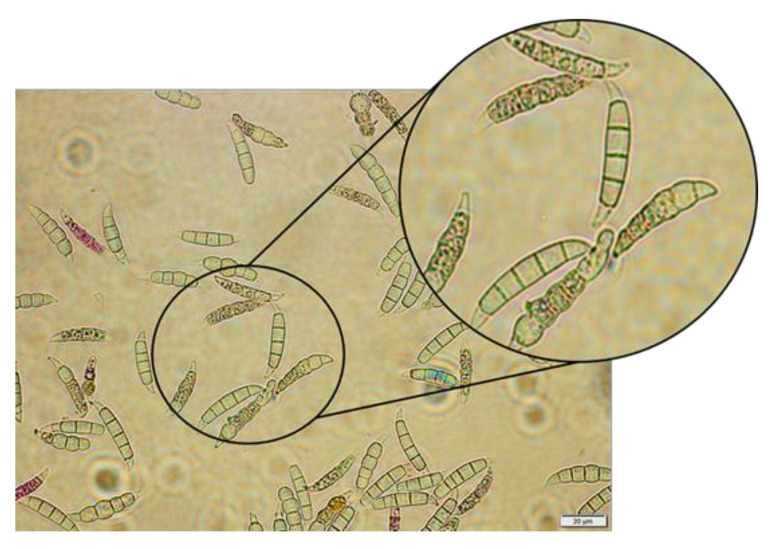
*Fusarium culmorum* 1913 treated with *Fucus vesiculosus* CCC Fraction 1 after 120 h of incubation; fraction concentration of 1.0%.

Macroconidia are generally more virulent than microconidia. The morphology of conidia, including their structural integrity, plays a crucial role in the virulence of *Fusarium* species, thus, any defragmentation or alteration in their structure could potentially reduce their virulence [71]. Successive subculturing of *Fusarium oxysporum* can lead to degeneration, which affects conidia production and virulence. Degenerated variants showed reduced virulence due to decreased activity of cell wall-degrading enzymes and lower expression of virulence-related genes. This suggests that structural changes in macroconidia, akin to defragmentation, could lead to a similar reduction in virulence [72]. Figure 8 presents *Fusarium culmorum* 1913 treated with *Fucus vesiculosus* CCC Fraction 1 (concentration of 1.0%) after 120 h of incubation with visible lysis and internal defragmentation of macroconidia segments as described by Pohl et al. [73].

### 2.4. The Activity of Plant Resistance Enzymes in Wheat Stems and Roots

**Figure 9:**
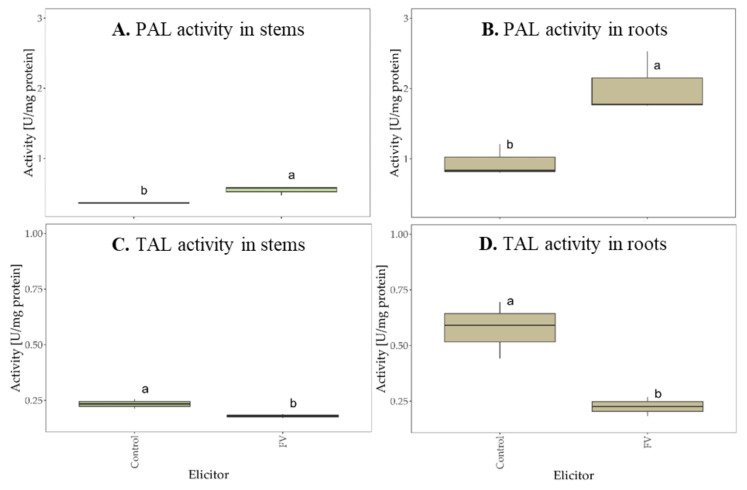
The activity of phenylalanine ammonia-lyase (PAL) and tyrosine ammonia-lyase (TAL) in wheat stems (**A**,**C**) and roots (**B**,**D**) after seeds inoculation with *Fucus vesiculosus* scCO_2_ extract (FV), compared to the water control (Control). The figures show the enzyme activity after 10 days of incubation (bars with different letters are statistically significantly different from each other, *p* < 0.05). SD was measured in three biological replicates (n = 3).

The potential applications of plant resistance enzymes in developing novel crop protection strategies are vast and promising. These enzymes play a crucial role in enhancing plant defenses against pathogens and pests, offering sustainable alternatives to traditional agrochemicals. By leveraging plant resistance enzymes, researchers aim to improve crop resilience, reduce chemical inputs, and promote environmentally friendly agricultural practices [74]. The studies conducted showed that *Fucus vesiculosus* scCO_2_ extract can induce plant immunity pathways by increasing the activity of the most important immune enzymes. The inoculation of wheat seeds with *Fucus vesiculosus* caused a significant increase in phenylalanine ammonia-lyase (PAL) activity in both the stems (Figure 9A) and roots (Figure 9B) of the plant, compared to the water control. The phenylalanine ammonia-lyase (PAL), a principal enzyme of the phenylpropanoid pathway, plays a crucial role in phytoalexin production and lignin biosynthesis [75]. However, the *Fucus vesiculosus* scCO_2_ extract did not significantly increase tyrosine ammonia-lyase (TAL) activity in wheat stems (Figure 9C) and roots (Figure 9D).

**Figure 10:**
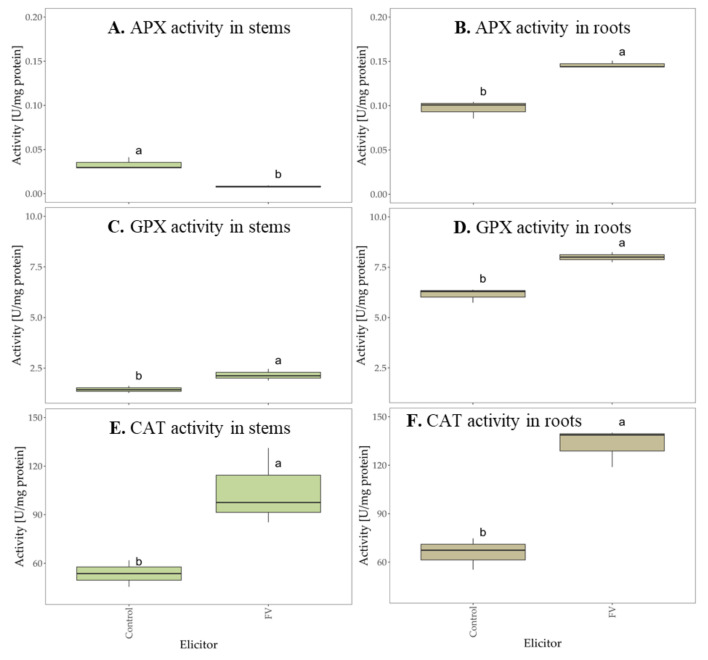
The activity of catalase (CAT), ascorbate (APX), and guaiacol (GPX) peroxidases in wheat stems (**A**,**C**,**E**) and roots (**B**,**D**,**F**) after seed inoculation with *Fucus vesiculosus* scCO_2_ extract (FV), compared to the water control (Control). The figures show the enzymes activity after 10 days of incubation (bars with different letters are statistically significantly different from each other, *p* < 0.05). SD was measured in three biological replicates (n = 3).

An increase in the activity of oxidative enzymes was also noted after the inoculation of wheat seeds with FV. Oxidative enzymes are produced by plant cells to protect against damage and oxidative stress [75]. They are the first line of defense to lower reactive oxygen species (ROS) levels in plant tissues [76]. The *Fucus vesiculosus* scCO_2_ extract caused a several-fold increase in APX, GPX, and CAT activity in wheat roots (Figure 10B,D,F). A significant increase in GPX and CAT activity was also noted in the plant stems (Figure 10C,E). The *Fucus vesiculosus* extract did not increase APX activity in wheat stems (Figure 10A).

Overall, the results obtained indicate that *Fucus vesiculosus* scCO_2_ extract may be used as an agent against plant phytopathogens with the ability to activate plant enzymes. The research by Circuncisão et al. [77,78] on *Fucus vesiculosus* ethanolic extracts elucidated the potential of indigenous seaweeds to be harnessed through comprehensive and sequential methodologies aimed at generating high-value alimentary products, thereby contributing to a circular and sustainable marine bioeconomy. The preliminary phase of the sequential extraction process devised for the brown macroalga *Fucus vesiculosus* possesses the capacity to influence the composition of the subsequent extracts and/or fractions. Another aspect worth mentioning is the relatively high extraction yields of *Fucus vesiculosus* water extraction resulting from 26 to 69 g per 100 g of dried seaweed [77]. However, supercritical carbon dioxide extracts offer a promising alternative to synthetic fungicides, providing an environmentally friendly option for managing fungal diseases in agriculture and postharvest handling [19,79]. On the other hand, higher concentrations of plant extracts containing secondary metabolites like polyphenols and flavonoids exhibit stronger fungistatic effects, highlighting the importance of optimizing the composition and concentration of plant extracts for effective plant protection against fungal pathogens [78]. In this manner, the combination of both scCO_2_ and water or ethanol extracts of *Fucus vesiculosus* may be a new effective approach in the search for plant growth biostimulants or agents combating pathogens.

## 3. Materials and Methods

### 3.1. Chemicals

Carbon dioxide (99.9%, *v*/*v*), which was used as the mobile phase in SFE, was stored in a CO_2_ installation tank. Authentic standards for chromatographic analyses (FAME Mix C_8_-C_24_, *cis*-5,8,11,14,17-eicosapentaenoic acid (EPA, 20:5*n-3*) and arachidonic acid (20:4*n-6*), as well as fucosterol, were purchased from Merck (Darmstadt, Germany). Trimethylsulfonium hydroxide solution in methanol for GC derivatization (TMSH solution), *tert*-butyl methyl ether (TBME), *n*-hexane, and methanol were purchased from Witko (Łódź, Poland).

Acetonitrile (ACN, >99%), *n*-hexane (>96%), and methanol (HPLC gradient grade, >99%) were purchased from Th. Geyer (Renningen, Germany). Benzotrifluoride (BTF, >99%) was supplied by Merck (Steinheim, Germany), whereas ethanol (>80%, distilled prior to use) was obtained from Carl Roth (Karlsruhe, Germany).

Reagents for the Reyes and Byrde (RB) medium (KH_2_PO_4_, MgSO_4_·7H_2_O, KCl, (NH_4_)_2_SO_4_, glucose, Na_2_B_4_O_7_·10H_2_O, FeSO_4_·7H_2_O, MnSO_4_·5H_2_O, (NH_4_)_6_Mo_7_O_24_·4H_2_O, ZnSO_4_·7H_2_O) were purchased from POCH (Gliwice, Poland) while CuSO_4_·5H_2_O was purchased from REACHIM (Lublin, Poland).

### 3.2. Samples

*Fucus vesiculosus* powder was purchased from the Zdrowsze Życie Company (Miejska Górka, Poland). The moisture content in the raw material of 8.12% was determined using a laboratory moisture analyzer (MAC 50/1/WH, RADWAG, Radom, Poland) at 20 °C. The powder (diameter < 0.5 mm) was used for the SFE process. Streptomycin was obtained from Polfa (Lublin, Poland). The fungal strain *Fusarium culmorum* 1913 (isolated from corn) was obtained from the fungi collection of the Plant Diseases Clinic and Bank of Pathogens of the Institute of Plant Protection—NRI in Poznań, Poland.

### 3.3. Instrumental Methods

#### 3.3.1. Supercritical Fluid Extraction (SFE)

The dynamic SFE process was performed in the Łukasiewicz Research Network—New Chemical Syntheses Institute (Puławy, Poland) on a half-technical scale SFE installation equipped with a 40 L extractor (Natex, Ternitz, Austria). The maximum operating temperature was up to 90 °C, while pressure was up to 1000 bar. Milled and sieved plant material (14.6 kg) was extracted with pure carbon dioxide. After reaching the desired temperature of 50 °C, the pressure was elevated to 500 bar followed by the outlet valve of the extractor opening and extract collecting in the separator at 40 °C and 50 bar. During the process, the scCO_2_ flow was maintained constantly at 220 kg/h (the CO_2_ was recycled). The SFE process was stopped when the CO_2_ consumption reached 80 kgCO_2_/kg feed. During extraction, a part of the moisture was separated from the plant material and collected with the extract. This moisture was removed from the extract before analysis using a rotary vacuum evaporator (Hei-VAP Precision, Heidolph Instruments, Schwabach, Germany). The SFE resulted in an extraction yield of 4.60 ± 0.32 wt.% calculated as the mass of extract obtained to the mass of the raw material extracted.

#### 3.3.2. Countercurrent Chromatography (CCC)

**Figure 11:**
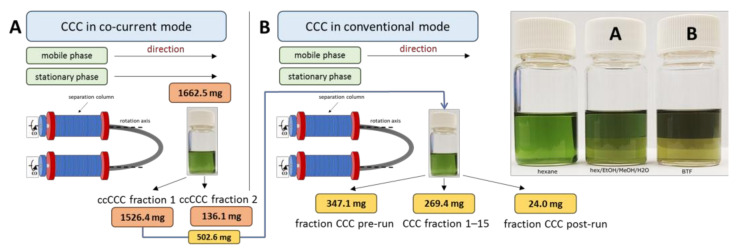
Schematic presentation of the two-step CCC separation ((**A**)—co-current mode (*n*-hexane/EtOH/MeOH/H*_2_*O); (**B**)—conventional mode (BTF)) and *Fucus vesiculosus* scCO_2_ extract in different solvent systems (BTF—benzotrifluoride; hex—*n*-hexane; EtOH—ethanol; MeOH—methanol; H*_2_*O—water) (on the right).

A QuikPrep MK8 instrument (AECS, Cornwall, UK), equipped with a flash 10 diode array detector (Ecom, Praha, Czech Republic) and a Gilson 203 B fraction collector, was used with a total coil volume of 236 mL and the maximum rotor speed of 870 rpm [80,81]. The system was operated in tail-to-head mode. Two separations were carried out, i.e., an enrichment step using CCC in co-current mode (ccCCC) followed by a final separation step using conventional CCC [53,56] (Table 4 and Table 5, Figure 11). The components of solvent systems were mixed according to the corresponding volume ratio in a 2.5 L separating funnel. After vigorous shaking and equilibration for ~30 min, the two phases were separated and degassed by ultrasonication and integration into the CCC system.

#### 3.3.3. Gas Chromatography with Flame Ionization Detection (GC/FID)

Fatty acids were determined after their conversion into methyl esters (FAME). A 7820A gas chromatograph (Agilent), equipped with a flame ionization detector (GC/FID), was used and data was analyzed using ChemStation C.01.03 Software (Agilent, USA). The temperature of the injector and detector was maintained at 250 °C and 300 °C, respectively. An HP-88 capillary column (100 m × 0.25 mm i.d., 0.25 μm film thickness, Agilent J&W) was installed in the GC oven which was kept for 4 min at 100 °C. Then, the temperature was linearly increased within 16 min to 200 °C (25 °C/min) and then at 5 °C/min to 250 °C which was held for 8 min (total run time: 34 min). The FAME C_8_–C_24_ standard (100 mg) was dissolved in 4 mL of TMBE/methanol mixture (2:1, *v*/*v*). *Fucus vesiculosus* carbon dioxide extract and CCC samples were prepared by dissolving each extract in 500 µL of TMBE/methanol mixture (2:1, *v*/*v*). After vortexing for 10 min, 250 µL of TMSH solution was added. The calibration curves of 20:5*n-3* and 20:4*n-6* were prepared individually. The samples were measured directly after derivatization in triplicate. The FAME peaks in extracts were identified by comparing their retention time with certified reference standards of FAME. The percentage relative of a particular fatty acid was calculated based on the peak area of the respective FAME to the total peak area of all the FAMEs detected by GC/FID.

#### 3.3.4. Gas Chromatography with Mass Spectrometry (GC/MS)

The qualitative analysis of *Fucus vesiculosus* scCO_2_ extract (transesterified with TMSH reagent) was performed with the use of a GC/MS system (Agilent, Santa Clara, CA, USA) equipped with tandem mass spectrometry. The separation of analyzed compounds was utilized with a DB-EUPAH (60 m × 0.25 mm × 0.25 μm) column with helium at the rate of 0.6 mL/min as a carrier gas. The initial oven temperature was set at 60 °C, followed by an increase to 310 °C at the ratio of 3 °C/min and isotherm of 310 °C until the end of the analysis. The components identification in a scan mode (*m*/*z* 35–*m*/*z* 650) was based on MassHunter software (C.01.03) with NIST Mass Spectral Library and literature data. The retention indices of compounds was in accordance with the analysis of *n*-alkanes (C8–C20 and C21–C40, Merck, Darmstadt, Germany).

#### 3.3.5. Supercritical Fluid Chromatography (SFC)

The fucosterol content in the supercritical carbon dioxide *Fucus vesiculosus* extract and CCC fractions was determined using a UPC^2^ system (Acquity Ultra-Performance Convergence Chromatography, Waters), equipped with an Acquity Fluoro Phenyl column (100 mm × 3.0 mm, 1.7 μm) at 40 °C, ABPR (back pressure regulator) at 126 bar, and a photodiode detector (PDA) at 220 nm. Both carbon dioxide and methanol were used as a mobile phase in a ratio of 98:2 (*v*/*v*) in an isocratic elution mode. Wavelength compensation was set in the range of 500–600 nm [32].

### 3.4. Antifungal Properties

The antifungal properties of CCC fractions were preceded by the preparation of 1 L sterilized Reyes and Byrde medium (121 °C, 0.75 bar, 30 min) [31,82]. In the next step, the mixture of microelements together with streptomycin was added.

The macroconidia of *Fusarium culmorum* 1913 strains used for the preparation of the inocula were obtained from the culture grown on Reyes and Byrde medium, with 1.0% glucose being a carbon source and cultivated in darkness at 20 °C and 60% relative humidity at 120 rpm for 7 days. Then, the fungal cultures were filtered through five layers of a sterile cotton gauze. The macroconidia were obtained by centrifuging the supernatants at 10,000 *g* for 15 min [31,76]. *Fucus vesiculosus* CCC fractions were prepared at concentrations of 0.05%, 0.2%, 0.5%, and 1.0%, similar to our previous study [31].

### 3.5. Wheat Growth and Enzyme Extraction

Seeds of winter wheat (*Triticum aestivum* L. cv Arkadia, Pińczyce, Poland) were surface sterilized using 0.1% HgCl_2_ at 120 rpm for 7 min. The seeds were washed five times with sterile distilled water at 120 rpm. Sterilized seeds were placed in glass Petri dishes with 20 places per plate, followed by incubation for 10 days at 20 °C. Subsequently, the stems were separated from the roots, and the fresh and dry weights and percentage of germinated seeds were determined. Then, 1 g of the plant parts was frozen in liquid nitrogen, and ground in a mortar to a powder. Next, 6 mL of extraction buffer (50 mM phosphate buffer (pH 7.5) containing 1 mM ethylenediaminetetraacetic acid (EDTA; Sigma-Aldrich, Steinheim, Germany), 1 mM phenylmethylsulfonyl fluoride (PMSF; Sigma-Aldrich, Steinheim, Germany), and 1% polyvinylpolypyrrolidone (PVPP poly(vinylpolypyrrolidone), Sigma-Aldrich, St. Louis, MO, USA) was added for 2 min. The extracts were filtered through a Miracloth filter (Merck Millipore, Darmstadt, Germany) and centrifuged (10,000 rpm at 4 °C for 15 min). The protein content of the resulting extracts was determined using the Bradford method [83], and the obtained extracts were frozen in liquid nitrogen and stored at -80 °C for future analyses [84,85].

### 3.6. Enzymes Activity

#### 3.6.1. Catalase (CAT) Activity

Catalase activity was determined via the decrease in H_2_O_2_ in the solution. Specifically, 950 µL of 20 mM H_2_O_2_ (POCH, Gliwice, Poland) in 50 mM phosphate buffer (pH 7.0) was added to 50 µL extract. The absorbance was measured spectrophotometrically (Jasco, Hachioji-shi, Tokyo, Japan) for 1 min at 240 nm. The activity was calculated using a molarity coefficient ε = 36 M/cm. Activity was expressed in U/mg protein [76,84].

#### 3.6.2. Ascorbic Peroxidase (APX) Activity

Ascorbate peroxidase activity was determined by the loss of added H_2_O_2_ in the presence of ascorbic acid. In brief, 400 µL 50 mM phosphate buffer (pH 7.0) and 250 µL 1 mM ascorbic acid (Sigma-Aldrich, Steinheim, Germany) were added to 100 µL extract. The reaction was initiated by adding 250 µL of 20 mM H_2_O_2_ (POCH, Gliwice, Poland). The absorbance was measured for 1 min at 290 nm spectrophotometrically. The activity was calculated using the molarity coefficient ε= 2.8 mM/cm. Activity was expressed in U/mg protein [84,86].

#### 3.6.3. Guaiacol Peroxidase (GPX) Activity

Guaiacol peroxidase activity was determined by the increment of tetraguaiacol in the presence of H_2_O_2_. For this purpose, 50 µL extract (20-fold diluted) was supplemented with 550 µL 100 mM phosphate buffer (pH 6.5), and 250 µL 60 mM guaiacol (Sigma-Aldrich, St. Louis, MO, USA). The reaction was initiated by adding 200 µL of 0.25% H_2_O_2_ (POCH, Gliwice, Poland). The absorbance was measured spectrophotometrically for 1 min at 470 nm. Activity was calculated using the molarity coefficient ε= 26.6 mM/cm. Activity was expressed in U/mg protein [84].

#### 3.6.4. Phenylalanine Ammonia-Lyase (PAL) Activity

Phenylalanine Ammonia-Lyase activity was determined by the transformation of L-phenylalanine into *trans*-cinnamic acid. In brief, 100 µL extract was supplemented with 500 µL 100 mM borate buffer (pH 8.8) and 400 µL 50 mM L-phenylalanine (Sigma-Aldrich, Steinheim, Germany). Absorbance was then measured at 290 nm, and samples were incubated at 37 °C for 1 h before the absorbance at 290 nm was measured again. The concentration of *trans*-cinnamic acid produced was calculated using a calibration curve, y=0.0081. Activity was expressed as U/mg of protein [86].

#### 3.6.5. Tyrosine Ammonia-Lyase (TAL) Activity

Tyrosine Ammonia-Lyase activity was determined by the transformation of L-tyrosine to *p*-coumaric acid. To 100 µL extract, 500 µL 100 mM borate buffer (pH 7.7) and 400 µL 10 mM L-tyrosine were added. Absorbance was then measured at 310 nm, and samples were incubated at 37 °C for 1 h before the absorbance at 310 nm was measured again. The concentration of *p*-coumaric acid produced was calculated using a calibration curve, y = 0.0137. Activity was expressed as U/mg of protein [86].

## 4. Conclusions

Brown seaweeds have been proven as viable agents in the fight against phytopathogens, attributable to their abundant bioactive compound composition, which provides environmentally sustainable alternatives to synthetic bactericides and fungicides. These marine algal species are endowed with a diverse array of metabolites that demonstrate considerable antimicrobial efficacy, rendering them effective against a variety of bacterial and fungal pathogens that impact plant health. The results obtained proved that *Fucus vesiculosus* possesses significant antifungal characteristics. For the first time, the investigation concentrated on the separation of *Fucus vesiculosus* supercritical carbon dioxide extract and the evaluation of the resultant fractions against serious phytopathogen, including *Fusarium*. Furthermore, an innovative attribute of *Fucus vesiculosus* supercritical carbon dioxide extract was examined, encompassing the activation of plant resistance enzymes.

## Figures and Tables

**Table 1:** Fatty acid and fucosterol content in *Fucus vesiculosus* raw material and scCO_2_ extract (obtained at 50 °C and 500 bar) using GC/FID.

Fatty Acids and Fucosterol, wt.%	Raw Material ^1^	scCO_2_ Extract
14:0	0.31 ± 0.10	9.3 ± 0.32
14:1*n-5*	0.01 ± 0.01	0.28 ± 0.01
16:0	0.44 ± 0.16	12.7 ± 0.09
16:1*n-7*	0.03 ± 0.02	1.05 ± 0.02
18:0	0.03 ± 0.01	1.0 ± 0.29
18:1*n-9*	1.1 ± 0.40	34.3 ± 0.02
18:2*n-6*	0.29 ± 0.10	9.8 ± 0.33
20:0	0.01 ± 0.01	0.47 ± 0.08
18:3*n-6*	0.08 ± 0.03	2.7 ± 0.04
20:1*n-9*	0.04 ± 0.01	1.7 ± 0.11
20:4*n-6*	0.22 ± 0.09	5.4 ± 0.13
20:5*n-3*	0.05 ± 0.02	1.6 ± 0.01
22:0	0.01 ± 0.01	0.27 ± 0.03
22:1*n-9*	0.03 ± 0.01	0.89 ± 0.05
SFA	0.81 ± 0.28	23.8 ± 0.54
MUFA	1.2 ± 0.13	38.2 ± 0.37
UFA	1.9 ± 0.21	57.7 ± 0.14
PUFA	0.64 ± 0.24	19.5 ± 0.73
Sum of all fatty acids	2.7 ± 0.91	81.5 ± 0.24
Fucosterol	0.13 ± 0.01	2.8 ± 0.65

^1^ based on accelerated solvent extraction (ASE) with *n*-hexane (extraction temperature 50 °C); scCO_2_—supercritical carbon dioxide; 14:0—myristic acid; 14:1*n-5*—myristoleic acid; 16:0—palmitic acid; 16:1*n-7*—palmitoleic acid; 18:0—stearic acid; 18:1*n-9—*oleic acid; 18:2*n-6—*linoleic acid; 20:0—arachidic acid; 20:1*n-9—cis*-11-eicosenoic acid; 18:3*n-6—*γ-linolenic acid; 20:4*n-6—*arachidonic acid; 20:5*n-3—*EPA; 22:0—behenic acid; 22:1*n-9—*erucic acid; SFA—saturated fatty acids; MUFA—monounsaturated fatty acids; UFA—unsaturated fatty acids; PUFA—polyunsaturated fatty acids.

**Table 2:** The fatty acid composition of *Fucus vesiculosus* scCO_2_ extract detected using GC/MS.

No.	Retention Time, min	% Area of Total ± SD	Chemical Compound	Base Peak (*m*/*z*)	Molecular Weight	Kovats Retention Index (KI)	Kovats Retention Index (KI) Based on Literature
1	12.92	9.5 ± 0.21	Myristic acid, methyl ester	74	242	1759	1725 [47]
2	13.17	0.19 ± 0.02	Myristoleic acid, methyl ester	55	240	1774	n.d.
3	14.37	0.28 ± 0.01	Pentadecanoic acid, methyl ester	74	256	1859	1825 [47]
4	16.05	14.0 ± 0.14	Palmitic acid, methyl ester	74	270	1949	1929 [47]
5	16.24	1.3 ± 0.09	Palmitoleic acid, methyl ester	55	268	1953	1909 [48]
6	18.14	0.23 ± 0.01	*cis*-10-Heptadecenoic acid, methyl ester	55	282	1991	n.d.
7	20.12	1.1 ± 0.02	Stearic acid, methyl ester	74	298	2139	2127 [47]
8	20.25	36.7 ± 0.67	Oleic acid, methyl ester	55	296	2143	2105 [48]
9	20.65	9.9 ± 0.37	Linoleic acid, methyl ester	67	294	2153	2066 [49]
10	20.79	0.41 ± 0.02	Unknown	n.a.	n.a.	2157	n.a.
11	21.34	1.9 ± 0.11	Linolenic acid, methyl ester	79	292	2151	2078 [50]
12	21.48	0.42 ± 0.02	Unknown	n.a.	n.a.	2174	n.a.
13	24.71	0.91 ± 0.18	Arachidic acid methyl ester	74	326	2341	2329 [47]
14	25.32	0.35 ± 0.05	11-Eicosenoic acid, methyl ester	55	324	2352	2279 [51]
15	25.47	5.6 ± 0.87	Arachidonic acid methyl ester	79	318	2354	2263 [48]
16	26.06	0.25 ± 0.01	Unknown	n.a.	n.a.	2365	n.a.
17	26.24	1.6 ± 0.06	*cis*-5,8,11,14,17-Eicosapentaenoic acid, methyl ester	79	302	2368	2232 [51]
18	29.47	0.44 ± 0.02	Behenic acid, methyl ester	74	354	2521	2531 [47]
19	29.94	0.85 ± 0.05	Unknown	n.a.	n.a.	2528	n.a.
20	30.94	0.29 ± 0.02	Unknown	n.a.	n.a.	2542	n.a.
21	33.41	0.57 ± 0.03	Unknown	n.a.	n.a.	2576	n.a.
22	34.18	0.72 ± 0.23	Methyl erucate	55	352	2587	2516 [50]
23	34.90	2.5 ± 0.19	Unknown	n.a.	n.a.	2597	n.a.
24	35.45	0.46 ± 0.04	Unknown	n.a.	n.a.	3009	n.a.
25	35.89	0.86 ± 0.05	Unknown	n.a.	n.a.	3069	n.a.
26	37.24	0.26 ± 0.03	Unknown	n.a.	n.a.	3109	n.a.
27	50.50	0.24 ± 0.02	Unknown	n.a.	n.a.	3177	n.a.
28	51.61	2.8 ± 0.17	Unknown	n.a.	n.a.	3183	n.a.
29	53.33	0.50 ± 0.03	Unknown	n.a.	n.a.	3192	n.a.
30	56.30	3.8 ± 0.09	Fucosterol	314	412	3273	n.d.
31	59.57	0.81 ± 0.19	Unknown	n.a.	n.a.	3285	n.a.
32	62.07	0.48 ± 0.05	Unknown	n.a.	n.a.	3294	n.a.

n.a.—not applicable; n.d.—no data.

**Table 3:** Quantitative analysis of *Fucus vesiculosus* CCC fractions using GC/FID (fatty acids) and SFC (UPC^2^ system; fucosterol).

Fatty Acids and Fucosterol, wt.%	Fraction Name																	
	ccCCC Fraction 1	Fraction CCC Pre-Run	Fraction CCC Post-Run	CCC Fraction 1	CCC Fraction 2	CCC Fraction 3	CCC Fraction 4	CCC Fraction 5	CCC Fraction 6	CCC Fraction 7	CCC Fraction 8	CCC Fraction 9	CCC Fraction 10	CCC Fraction 11	CCC Fraction 12	CCC Fraction 13	CCC Fraction 14	CCC Fraction 15
14:0	9.2 ± 0.46	8.5 ± 0.32	12.3± 0.24	4.0 ± 0.15	3.7 ± 0.19	4.5 ± 0.54	5.5 ± 0.15	4.3 ± 0.08	4.1 ± 0.02	5.0 ± 0.43	5.1 ± 0.32	3.9 ± 0.15	3.4 ± 0.02	3.3 ± 0.03	5.5 ± 0.11	5.7 ± 0.24	9.1 ± 0.21	6.1 ± 0.22
14:1*n-5*	0.23 ± 0.01	0.22 ± 0.02	0.12 ± 0.01	0.24 ± 0.02	0.00 ± 0.00	0.00 ± 0.00	0.00 ± 0.00	0.00 ± 0.00	0.00 ± 0.00	0.00 ± 0.00	0.00 ± 0.00	0.00 ± 0.00	0.00 ± 0.00	0.00 ± 0.00	0.00 ± 0.00	0.00 ± 0.00	0.00 ± 0.00	0.00 ± 0.00
16:0	11.4 ± 0.44	13.3 ± 0.74	6.5 ± 0.31	6.3 ± 0.23	7.3 ± 0.99	8.0 ± 0.44	8.9 ± 0.18	10.0 ± 0.04	12.8 ± 0.05	11.9 ± 0.02	6.8 ± 0.32	3.4 ± 0.14	3.0 ± 0.02	3.5 ± 0.04	7.0 ± 0.12	12.5 ± 0.55	14.3 ± 0.97	12.1 ± 0.17
16:1*n-7*	1.2 ± 0.02	1.2 ± 0.05	1.5 ± 0.04	0.94 ± 0.05	0.75 ± 0.02	0.68 ± 0.07	0.72 ± 0.05	0.65 ± 0.03	0.58 ± 0.09	0.71 ± 0.07	0.76 ± 0.06	0.65 ± 0.04	0.57 ± 0.03	0.55 ± 0.02	0.67 ± 0.04	0.86 ± 0.09	1.03 ± 0.04	1.23 ± 0.03
18:0	1.0 ± 0.02	1.2 ± 0.03	0.65 ± 0.03	0.57 ± 0.02	0.66 ± 0.03	1.0 ± 0.02	1.5 ± 0.05	1.2 ± 0.09	0.81 ± 0.02	0.62 ± 0.03	0.39 ± 0.03	1.1 ± 0.09	0.97 ± 0.01	0.22 ± 0.01	0.30 ± 0.02	0.43 ± 0.02	0.52 ± 0.11	0.42 ± 0.20
18:1*n-9*	31.8 ± 0.33	38.9 ± 1.42	9.2 ± 0.36	12.6 ± 0.36	13.9 ± 0.99	16.3 ± 0.82	18.5 ± 0.43	21.2 ± 0.54	31.0 ± 0.09	35.4 ± 1.34	21.7 ± 1.02	10.0 ± 0.05	8.7 ± 0.99	7.1 ± 1.29	11.3 ± 1.52	18.6 ± 1.85	21.1 ± 2.01	18.1 ± 1.67
18:2*n-6*	9.4 ± 1.08	10.1 ± 0.33	8.6 ± 0.17	9.7 ± 0.38	6.4 ± 0.54	5.2 ± 0.23	5.3 ± 1.01	5.0 ± 1.32	4.3 ± 0.04	4.5 ± 0.14	4.7 ± 0.03	4.4 ± 0.85	3.9 ± 1.08	4.6 ± 0.44	6.1 ± 0.51	7.8 ± 0.92	9.0 ± 1.43	8.8 ± 1.11
20:0	0.46 ± 0.03	0.00 ± 0.00	0.15 ± 0.02	0.35 ± 0.09	0.63 ± 0.04	0.60 ± 0.01	0.51 ± 0.02	0.32 ± 0.01	0.23 ± 0.01	0.17 ± 0.02	0.12 ± 0.03	0.06 ± 0.01	0.06 ± 0.01	0.09 ± 0.02	0.19 ± 0.03	0.56 ± 0.11	0.64 ± 0.27	0.60 ± 0.13
18:3*n-6*	0.78 ± 0.02	0.40 ± 0.08	4.6 ± 0.41	1.5 ± 0.07	0.25 ± 0.04	3.8 ± 0.03	3.1 ± 0.02	1.9 ± 0.01	1.3 ± 0.12	1.1 ± 0.43	0.77 ± 0.02	0.49 ± 0.03	2.5 ± 0.02	0.7 ± 0.02	1.2 ± 0.14	2.3 ± 0.26	1.0 ± 0.03	2.3 ± 0.02
20:1*n-9*	2.1 ± 0.03	2.2 ± 0.29	2.3 ± 0.24	7.9 ± 0.16	5.1 ± 0.15	3.6 ± 0.21	3.0 ± 0.23	1.8 ± 0.17	1.2 ± 0.09	1.0 ± 0.05	0.77 ± 0.02	0.49 ± 0.03	0.44 ± 0.04	0.66 ± 0.03	1.2 ± 0.02	2.1 ± 0.05	2.6 ± 0.09	2.1 ± 0.04
20:4*n-6*	4.9 ± 0.19	5.0 ± 0.25	7.0 ± 0.57	14.4 ± 0.92	9.6 ± 0.27	6.4 ± 0.46	5.1 ± 0.16	3.3 ± 0.15	2.8 ± 0.07	2.3 ± 0.08	1.6 ± 0.02	1.0 ± 0.09	0.8 ± 0.11	1.7 ± 0.16	0.21 ± 0.05	7.1 ± 0.99	5.9 ± 0.33	4.4 ± 0.64
20:5*n-3*	1.2 ± 0.02	0.97 ± 0.10	2.0 ± 0.24	7.9 ± 0.28	6.0 ± 1.02	4.1 ± 0.99	3.3 ± 0.04	2.2 ± 0.05	1.6 ± 0.11	1.1 ± 0.05	0.74 ± 0.09	0.41 ± 0.05	0.36 ± 0.03	0.28 ± 0.03	3.3 ± 0.02	0.58 ± 0.01	1.0 ± 0.09	0.95 ± 0.01
22:0	0.30 ± 0.02	0.00 ± 0.00	0.00 ± 0.00	0.24 ± 0.05	0.38 ± 0.02	0.12 ± 0.01	0.11 ± 0.02	0.07 ± 0.01	0.11 ± 0.01	0.13 ± 0.02	0.07 ± 0.01	0.04 ± 0.01	0.04 ± 0.01	0.17 ± 0.01	0.38 ± 0.01	0.11 ± 0.01	0.29 ± 0.02	0.12 ± 0.02
22:1*n-9*	0.12 ± 0.01	0.08 ± 0.02	0.16 ± 0.03	0.08 ± 0.02	0.09 ± 0.01	0.00 ± 0.00	0.00 ± 0.00	0.00 ± 0.00	0.00 ± 0.00	0.00 ± 0.00	0.00 ± 0.00	0.00 ± 0.00	0.00 ± 0.00	0.00 ± 0.00	0.00 ± 0.00	0.00 ± 0.00	0.00 ± 0.00	0.14 ± 0.01
SFA	22.3 ± 0.57	22.9 ± 0.79	19.5 ± 0.94	11.5 ± 0.27	12.6 ± 0.15	14.3 ± 0.12	16.5 ± 0.62	16.0 ± 0.15	18.1 ± 0.29	18.1 ± 0.11	12.5 ± 0.19	8.5 ± 0.22	7.5 ± 0.93	7.30 ± 0.09	13.3 ± 1.45	19.3 ± 2.56	24.8 ± 2.22	19.4 ± 1.77
MUFA	35.5 ± 0.05	42.6 ± 0.86	13.3 ± 0.65	21.8 ± 0.16	19.8 ± 0.45	20.6 ± 2.09	22.2 ± 1.11	23.7 ± 0.98	32.8 ± 0.33	37.1 ± 1.04	23.2 ± 1.26	11.1 ± 0.55	9.7 ± 0.49	8.3 ± 0.29	13.2 ± 0.18	21.6 ± 0.87	24.7 ± 1.06	21.6 ± 1.39
UFA	51.7 ± 0.52	59.1 ± 1.45	35.5 ± 0.51	55.3 ± 1.02	42.1 ± 1.99	40.1 ± 1.54	39.0 ± 2.9	36.1 ± 2.43	42.8 ± 2.67	46.1 ± 1.55	31.0 ± 1.44	17.4 ± 1.32	17.3 ± 1.82	15.6 ± 1.09	24.0 ± 1.04	39.3 ± 0.56	41.6 ± 0.78	38.0 ± 0.49
PUFA	16.3 ± 0.32	16.5 ± 0.49	22.2 ± 0.81	33.5 ± 0.93	22.3 ± 0.94	19.5 ± 0.33	16.8 ± 0.67	12.3 ± 1.08	9.9 ± 0.56	9.0 ± 0.77	7.7 ± 0.99	6.3 ± 1.03	7.6 ± 0.33	7.3 ± 0.58	10.9 ± 1.03	17.8 ± 1.11	16.8 ± 1.76	16.4 ± 2.02
FAME	74.0 ± 3.24	82.0 ± 2.04	55.0 ± 1.75	66.7 ± 0.22	48.8 ± 0.93	50.1 ± 1.44	51.7 ± 1.94	49.4 ± 0.95	59.1 ± 0.29	62.5 ± 2.45	42.1 ± 1.92	24.8 ± 1.54	23.8 ± 1.99	21.8 ± 2.39	35.4 ± 3.01	55.7 ± 2.27	62.7 ± 1.92	53.9 ± 1.74
Fucosterol	4.9 ± 0.15	n.i.	0.72 ± 0.05	n.i.	n.i.	n.i.	n.i.	n.i.	n.i.	4.8 ± 0.03	27.8 ± 0.58	59.2 ± 1.09	67.0 ± 1.31	79.4 ± 0.99	39.9 ± 0.82	13.8 ± 0.15	3.1 ± 0.23	n.i.

14:0—myristic acid; 14:1*n-5*—myristoleic acid; 16:0—palmitic acid; 16:1*n-7*—palmitoleic acid; 18:0—stearic acid; 18:1*n-9—*oleic acid; 18:2*n-6—*linoleic acid; 20:0—arachidic acid; 20:1*n-9—cis*-11-eicosenoic acid; 18:3*n-6*—γ-linolenic acid; 20:4*n-6—*arachidonic acid; 20:5*n-3—*EPA; 22:0—behenic acid; 22:1*n-9—*erucic acid; SFA—saturated fatty acids; MUFA—monounsaturated fatty acids; UFA—unsaturated fatty acids; PUFA—polyunsaturated fatty acids; FAME—fatty acids methyl esters; n.i.—not identified; ccCCC—CCC operated in co-current mode.

**Table 4:** Countercurrent chromatography in co-current mode (ccCCC) of *Fucus vesiculosus* scCO_2_ extract; *n*-hexane/ethanol/methanol/water system (34:11:12:2, *v*/*v*/*v*/*v*) (initial attempt).

Time, min:s	Upper Phase (Initial Mobile), %	Lower Phase (Initial Stationary), %	Elution Mode Valve Position	Flow Rate, mL/min	Rotation Speed, RPM
0:00	0	100	tail-to-head	10	0
24:00	0	100	tail-to-head	10	0
36:00	100	0	tail-to-head	2	870
46:00	33	67	tail-to-head	6	870
99:00	33	67	tail-to-head	6	870
152:00	33	67	tail-to-head	6	870

0–24 min: coil filling; 24–36 min: S_f_ (retention of the stationary phase) determination; 36–46 min: equilibration in co-current mode; 46–99 min: first co-current separation; 99–152 min: second co-current separation; RPM—revolutions per minute.

**Table 5:** Countercurrent chromatography of *Fucus vesiculosus* scCO_2_ extract; *n*-hexane/BTF/ACN (20:7:13, *v*/*v*/*v*) (final separation attempt).

Time, min:s	Upper Phase (Initial Mobile), %	Lower Phase (Initial Stationary), %	Elution Mode Valve Position	Flow Rate, mL/min	Rotation Speed, RPM
0:00	0	100	tail-to-head	10	0
24:00	0	100	tail-to-head	10	0
47:30	100	0	tail-to-head	2	870
147:30	100	0	tail-to-head	2	870

0–24 min: coil filling; 24–47:30 min: S_f_ (stationary phase retention) determination; 47:30–147:30 min: conventional countercurrent chromatography; BTF—benzotrifluoride; ACN—acetonitrile; RPM—revolutions per minute.

## Data Availability

Data will be made available on request.
